# Prediction and Detection of Localised Corrosion Attack of Stainless Steel in Biogas Production: A Machine Learning Classification Approach

**DOI:** 10.3390/ma18051057

**Published:** 2025-02-27

**Authors:** María Jesús Jiménez-Come, Francisco Javier González Gallero, Pascual Álvarez Gómez, Victoria Matres

**Affiliations:** 1 Escuela Técnica Superior de Ingeniería de Algeciras, Universidad de Cádiz, Avda. Ramón Puyol s/n, 11202 Algeciras, Cádiz, Spain; javier.gallero@uca.es (F.J.G.G.); pascual.alvarez@uca.es (P.Á.G.); 2 ACERINOX, Polígono Industrial Palmones, 11379 Los Barrios, Cádiz, Spain

**Keywords:** localised corrosion, biogas, stainless steel, machine learning

## Abstract

Biogas contributes to environmental protection by reducing greenhouse gas emissions and promoting the recycling of organic waste. Its utilization plays a crucial role in addressing the challenges of climate change and sustainability. However, the deterioration of process plants involved in biogas production due to corrosion has a critical impact on the safety and durability of their operations. In order to maintain the safety of structures in terms of service life with respect to corrosion, it is essential to develop effective corrosion engineering control methods. Electrochemical techniques have become a useful tool by which to evaluate corrosion resistance. However, these techniques may require microscopic analysis of the material surface and the analysis may be influenced by subjective factors. To solve this drawback, this work proposes the use of SVM models to predict the corrosion status of the material used in biogas production with no need to perform microscopic analysis after the electrochemical test. The obtained results of sensitivity and specificity equal to 0.94 and 0.97, respectively, revealed the utility of the proposed stochastic models to assure the corrosion state of the equipment involved in biogas production. SVM-based models are an effective alternative for accurately evaluating material durability and comparing the corrosion resistance of different materials in biogas environments. This approach facilitates the selection of the most suitable material to achieve greater durability and long-term performance. Synopsis: The results show that the proposed model is a useful tool to predict the behaviour of stainless steel against corrosion according to the environmental conditions to which the material is exposed in biogas production.

## 1. Introduction

The uncontrolled emissions of greenhouse gas (GHG) generated by agricultural activities have an influence on technical, economic, and social factors that can affect the sustainability of these activities [1]. With the aim of achieving the goals of the Paris Agreement and to mitigate the impact of climate change, the EU has proposed a reduction in GHG emissions by 40% by 2030 compared to 1990 [2]. In addition, the Sustainable Development Goals (SDGs) have been set by the United Nations to reduce worldwide waste generation by 2030 [3]. One option to reduce global warming and transform the pollutant waste into a valuable resource is through the production of biogas. This process involves the conversion of biodegradable material, in the absence of oxygen, to biogas (ca. 55%vol CH_4_, 45%vol CO_2_) as a result of different microbial processes [4]. Biodigesters are considered an environmentally friendly technology and suitable for a decentralised application, being easy to construct and operate. Most sustainability studies related to biogas and other renewable energy systems have focused on limited aspects, such as feedstock sustainability or energy efficiency [5]. However, it naturally follows that any evaluation of the durability of the systems used in the production process would be an important point to analyse. Like most industrial assets, some elements of a biogas plant can be affected by corrosion, since the oxidation and fermentation processes in digesters, whether concrete or steel, provide a perfect environment for corrosion. In order to maintain the safety of structures in terms of their service life with respect to corrosion in biogas production, monitoring and design are important measures [6,7].

The deterioration of metals and alloys as the result of their interactions with the surrounding environment is called corrosion. Corrosion is a worldwide crucial problem that strongly affects natural and industrial environments. This degradation may cause weakness in the material due to the reduction of its area, changes in the crystalline structure, and reduced strength, leading to toxic substance leakage, equipment structure failure, or even explosion accidents [8,9]. Although biogas technology is quite mature, the problems related to corrosion during its production are a topic that has not been well documented. In biogas production, the biodigesters are exposed to direct contact with a wide range of aggressive products that may cause damage to their durability [10]. Therefore, in order to prevent failures in the system, it is necessary to protect the biodigesters from corrosion attack, reducing the maintenance tasks at the same time.

Concrete is widely used for biogas production tanks, but its large-scale construction requires heavy machinery, and corrosion prevention remains challenging even with special coatings [11]. In contrast, stainless steel has emerged as a suitable alternative due to its durability, corrosion resistance, mechanical strength, and recyclability [12]. Its extensive use spans industries such as agriculture, transport, and chemical engineering [13,14]. The choice of stainless steel grade depends on functional and economic factors, making material selection critical for biodigester longevity.

The corrosion resistance of stainless steel is attributed to its passive film, formed by chromium reacting with oxygen. A minimum of 10.5% chromium is required for passivation, but aggressive environments can compromise this layer, leading to corrosion [15]. While uniform corrosion is predictable and manageable, localised corrosion, particularly pitting, is more severe and unpredictable, resulting in significant risks to industrial applications [16,17].

With the rapid development of biogas production, the problems related to corrosion have become increasingly important, making corrosion management a crucial factor to ensure process safety. For the industrial sector, the global cost of corrosion was estimated to be 3.4% of the global GDP [18]. It is estimated that savings of between 15 and 35% of the cost of corrosion could be realised by using available corrosion control practices [19]. The prediction of corrosion behaviour, as the preliminary stage of equipment corrosion management, is a crucial step to ensure proper corrosion control and the safety protection of the material [20]. However, the highly nonlinear nature of corrosion and the lack of a theoretical basis for some corrosion phenomena in certain environments have promoted the development of models based on artificial intelligence techniques being applied to the study of corrosion phenomena [21,22,23].

During the last few years, artificial intelligence techniques have been applied to model complex real problems based on experimental data [24,25,26,27,28,29,30,31,32,33,34,35]. Scientists have made great efforts to obtain effective tools to model corrosion behaviour. Among the different artificial intelligence approaches that can be found in the literature, one of the best known is Support Vector Machines (SVMs). These techniques have been proposed by many authors for structural reliability analysis [36,37]. However, research about corrosion modelling using SVMs is scarce. Wen et al. [38] proposed an SVM model to predict the corrosion rate of carbon steel in different seawater conditions. Hatamy et al. [39] applied the SVM technique to predict the CO_2_ corrosion rate in oil and gas industries, considering different parameters such as CO_2_ partial pressure, temperature, pH, and flow velocity. Chou et al. [22] applied SVM to predict the pitting corrosion risk of steel-reinforced concrete and the marine corrosion rate of carbon steel. Lv et al. [40] used SVMs to calculate different cross-section digitalisation parameters to predict the sectional corrosion rate of steel, and Seghier et al. [41] proposed an SVM model to model a pitting corrosion attack in oil and gas pipelines. Despite the fact that this technique has been used by some authors to attain further knowledge about the corrosion behaviour of different materials, limited instances in the literature can be found for those environments related to biogas production.

In this study, according to the experience of the authors and the good results obtained with the SVM models developed in previous studies [42,43,44], the use of an SVM was proposed to develop a stochastic model capable of predicting the behaviour of localised corrosion of different types of stainless steels involved in biogas production. Compared with the manual classification of corrosion resistance of the different materials carried out through electrochemical tests and microscopic analysis, the classification speed resulting from the SVM technology is faster and can reduce the impact of subjectivity on the results. As far as we are aware, no previous study in the scientific literature has employed SVM models to analyse the corrosion behaviour of stainless steels in biogas environments, highlighting the novelty and significance of this research in the field. The results demonstrate the great usefulness of the proposed model, which becomes a valuable tool in biodigester design by accurately predicting the corrosion behaviour of stainless steel under specific environmental conditions in biogas production. The developed models represent a great advance in this field since they not only allow economic savings related to maintenance tasks but also improve the durability of the equipment involved in biogas production, avoiding problems such as pollution, leaks, and product loss.

## 2. Experimental Procedure

As previously introduced, with the development of computer technology, machine learning technology has been adopted to address classification and recognition problems. In this case, the SVM technique was proposed to model the localised corrosion behaviour of different grades of stainless steel used for equipment manufacturing involved in biogas production. The model was used to predict the corrosion status of the material based on electrochemical tests with no need to perform microscopic analysis. The compositions of different grades of stainless steel considered in this study are presented in the following Table 1:

The main objective of this study was to predict the corrosion behaviour of the material according to the environmental conditions to which it may be exposed in biogas production in order to minimise the impact of corrosion on this material and to prevent equipment durability problems. For this reason, the experiments were carried out under different conditions by using artificial solutions (AS) simulating biogas environments. The compositions of the artificial solutions tested in this study are collected in the following Table 2:

The experimental dataset was obtained by means of electrochemical tests in order to evaluate the susceptibility of different grades of stainless steel to suffer localised attacks in biogas environments. Two types of localised corrosion were analysed since they are the most common attacks in these environments: pitting and crevice corrosion.

**Figure 1:**
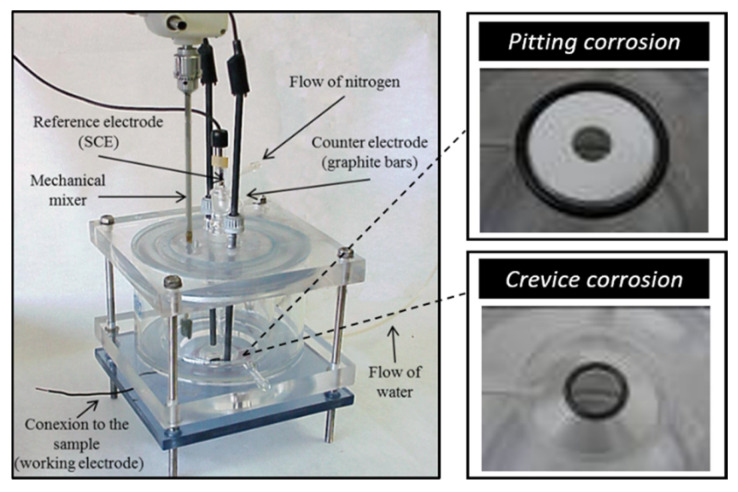
Design of the electrochemical cell used to evaluate the localised corrosion resistance of different grades of stainless steel in biogas environments: pitting and crevice corrosion.

The electrochemical tests were based on ASTM standards G3-94, “Conventions Applicable to Electrochemical Measurements in corrosion testing”, and G5-94, “Making potentiostatic and Potentiodynamic Anodic Polarization Measurements” [45]. In this application, a variable potential was applied to the sample under study, located in the cell shown in Figure 1, and the current density of the system was measured for each potential value. The potential was represented versus the current density to obtain the polarisation curve for the sample, providing information about the behaviour of the material under the tested conditions. According to the polarisation curve, a parameter called “breakdown potential” can be defined as an indicator of the susceptibility to the initiation of localised corrosion. This parameter is defined by the potential where an abrupt increase in the anodic current density is observed. Many authors have determined that this condition can be evaluated as the potential at which the current density exceeds 100 µA/cm^2^ [46]. However, there are materials with high corrosion resistance, and for these cases, the transpassive region can be reached, and the breakdown of the passive layer is not caused by localised corrosion. Therefore, it is necessary to analyse the material surface microscopically after electrochemical testing in order to determine if there is a pit or crevice on the surface of the material, or, on the contrary, if the material is resistant to localised corrosion. In this experimental work, according to the microscopic analysis, when any evidence of localised corrosion was observed on the surface, the sample was defined as a corrosion pattern. With the aim of ensuring the reproducibility of the experimental results, each tested condition was repeated three times. A total of 520 groups of the experimental dataset were recorded. Table 3 shows the detailed information of the experimental conditions.

According to the type of localised corrosion studied, pitting or crevice corrosion, the electrochemical cell was prepared with different configurations. When the pitting corrosion test was carried out, the crevice formation was avoided by using the inner and outer o-rings, as shown in Figure 1. For the pitting corrosion test, a paper filter was located between the o-rings to carry the flow of water into the cell. For the crevice corrosion test, the system contained an inner o-ring in contact with the sample to be analysed, promoting the formation of a crevice.

Based on the electrochemical test, each sample was defined according to the following features: the chemical composition of the alloy, the properties of the artificial solution that simulated the biogas environment (the type of artificial solution and temperature), the material surface finish (according to the type of polishing used for the sample: no polishing or #600# grain polishing), and the type of localised corrosion analysed (pitting or crevice corrosion). All of these features, in addition to the breakdown potential evaluated from the polarisation tests, were considered as inputs for the proposed SVM model, whereas the output was defined according to the corrosion status of the sample analysed under a microscope (1 for the corrosion sample when the corrosion attack was observed on the material surface and 0 otherwise). Up to 520 groups of the experimental dataset were recorded.

The proposed SVM model attempted to predict the corrosion status of the material under study for new experimental conditions that had not been previously evaluated. In this case, after subjecting the sample to electrochemical testing, the surface status of the sample could be predicted by the model without the need to resort to microscopic analysis. Compared with manual classification, the classification speed when using the proposed model is faster, and it can reduce the impact of subjectivity on the results, as the detection of a pit or crevice on the surface of the material, after electrochemical testing, is a complex task and may depend on experience and human behaviour.

## 3. Methodology

The Support Vector Machine theory was developed by Vapnik [47]. This technique has become more popular due to its many attractive features, one of which is its promising empirical performance. SVMs are based on the structural risk minimisation principle, which has been demonstrated to be superior to the traditional empirical risk minimisation principle (ERM) used by artificial neural networks [48]. This is due to the great ability shown by SVMs to generalise, since this technique tries to minimize an upper bound of the expected risk instead of focusing on minimizing the errors evaluated in the training data, which is the specific feature of ERM. SVM can be applied to solve classification and regression problems.

**Figure 2:**
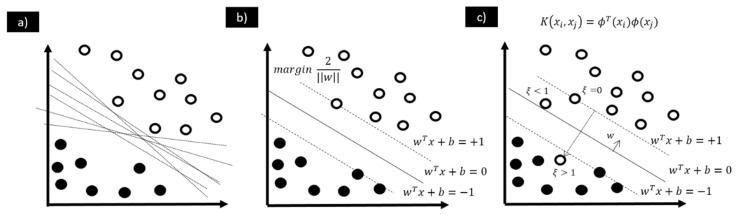
Examples of SVM models for binary classification model: (**a**) hyperplanes for linearly separable case, (**b**) SVM description for linearly separable case, and (**c**) soft margin SVM classification model.

For classification problems, the goal is to find a function capable of separating the different classes presented to the model. According to Figure 2a, there may be different linear hyperplanes that can separate the data from the different classes. However, there is only one classifier that maximises the margin; that is, the distance between the hyperplane and the nearest patterns of each class. This frontier is called the optimal separating hyperplane. This hyperplane can be used to classify new data.

For binary classification problems, the system can be defined according to the following equation:

(1) $$D=\left\{\left(x_{1},y_{1}\right),\;\ldots ,\left(x_{l},y_{l}\right)\right\}\;\;\;for\;i=1,\ldots ,\;l\;x\;\in {\mathbb{R}}^{l},\;\;\;\;\;y\in \left\{-1,1\right\}$$

where the hyperplane is defined by:

(2) 〈w,x〉+b=0

In this sense, the set of patterns can be optimally separated when there is a solution that defines the frontier between the different classes without error and provides the maximum distance between the hyperplane and the closest pattern of each class; see Figure 2b. For this case, Vapnik defines the optimisation problem to be solved as:

(3) $$\min\left|\langle w,x_{i}\rangle +b\right|=1$$

with the following constraints:

(4) $$y_{i}\left[\langle w\cdot x_{i}\rangle +b\right]\geq 1\;\;\;\;for\;\;\;\;i=1,\ldots ,l$$

The optimisation problem is solved considering the Karush–Kuhn–Tucker (KKT) conditions and using Lagrange multipliers [49]. The patterns whose Lagrange multipliers are non-zero are considered as support vectors, and they are the points that contain the information to define the optimal hyperplane to solve the classification problem. When the problem is linearly separable, all the support vectors are located on the margin, and in this case, the number of support vectors is reduced. However, when the training data are not linearly separable, see Figure 2c, additional variables related to misclassification, ξ_i_, are introduced in the optimisation problem [50]. For a non-linearly separable case, the constraints are modified to be defined as follows:

(5) $$y_{i}\left[\langle w,x_{i}\rangle +b\right]\geq 1-\xi_{i}\;\;\;\;for\;\;\;\;i=1,\ldots ,l$$

where *ξ_i_* are the slack variables with non-negative values. For this case, the optimal separating hyperplane is determined by:

(6) $$\phi \left(w,\xi \right)=\frac{1}{2}\;{\left|\left|w\right|\right|}^{2}+C\sum_{i}\xi_{i}$$

where *C* is related to the regularisation parameter introduced to define a balance between maximising the margin and minimising the classification error [51]. In addition, when a linear boundary is inappropriate for the problem under study, the input vector can be mapped into a high-dimensional feature space by using kernel functions, where the SVMs can define an optimal separating hyperplane [52]. Among the acceptable kernel functions, the most frequently used are polynomials (SVM-POL) and radial basis functions (SVM-RBF).

Apart from selecting the best kernel function, one of the most critical steps in these classification problems is how to define the regularisation parameter, C. In this study, a cross validation technique is applied to determine the optimal value of this parameter and those involved in kernel functions: the polynomial degree for polynomial function and the gamma parameter for radial basis function kernel. To ensure good generalisation capability and to avoid overfitting, the experimental dataset, consisting of 90 patterns, was randomly divided into 2 subsets using the 5-fold cross-validation method: training and validation sets. In each iteration, the model was trained on 80% of the original patterns and evaluated on the remaining 20%. This process was repeated 20 times, resulting in a total of 100 training runs (5 folds × 20 repetitions). The classification performance was measured based on the validation set, an independent subset not previously presented to the model. This process was implemented in MATLAB^®^.

Finally, the normalisation of the original dataset can be required for certain kernel functions due to their restricted domain. For this reason, all the original variables were normalised in the range [−1, 1].

**Figure 3:**
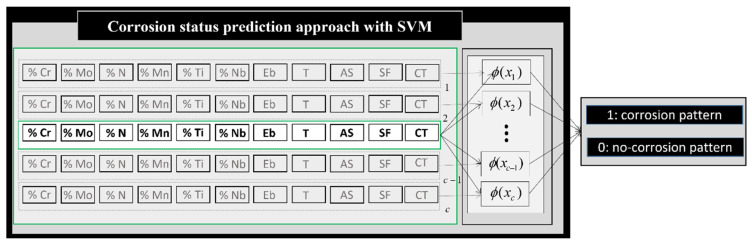
Structure of the support vector machine model proposed for corrosion status prediction of stainless steel in a biogas environment. The training patterns, defined by stainless steel composition, breakdown potential (Eb), temperature (T), type of artificial solution (AS), surface finish (SF), and corrosion type analysed (CT: pitting or crevice corrosion), are mapped into a feature space with the function φ. The output corresponds to the corrosion status of the test pattern: 1 for corrosion status and 0 otherwise.

In this way, the SVM model was proposed to determine the corrosion status of the sample without the need to carry out microscopic analysis for new conditions that have not been tested before (see Figure 3). This study can be considered a classification problem; according to the sample features and the environmental conditions, the proposed SVM model will have the capacity of determining the corrosion resistance of the material under different environmental conditions involved in biogas production.

For the classification problem, the performance of the proposed model can be evaluated according to precision and accuracy indices defined by the following equations:

(7) $$\mathrm{Precision}=TPR=\frac{TP}{TP+FP}$$

(8) $$\mathrm{Accuracy}=\frac{TP+TN}{P+N}$$

where TP (true positive) is defined as the number of corrosion patterns that have been classified correctly and TN (true negative) is the number of no-corrosion patterns that have been classified correctly, whereas FP (false positive) is the number of those no-corrosion patterns that have been misclassified as corrosion patterns and FN (false negative) is the number of corrosion patterns that have been misclassified as no-corrosion patterns. P and N correspond to the number of corrosion and no-corrosion patterns from the original data, respectively.

In addition, for the classification problem, sensitivity and specificity can be defined by the following equations:

(9) $$\mathrm{sensitivity}=\frac{TP}{TP+FN}$$

(10) $$\mathrm{specificity}=1-FPR=\frac{TN}{TN+FP}$$

**Figure 4:**
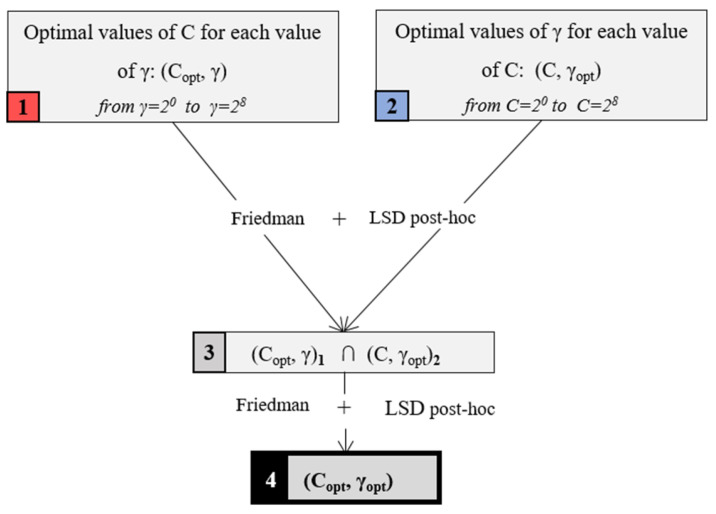
Flow chart of the multiple comparison analysis for the SVM-RBF models.

With the aim of determining the optimal configuration for the proposed SVM model, multiple comparison analysis was applied. In this study, the Friedman test was considered, as it is usually referred to as one of the most important non-parametric tests for this type of analysis [53]. When the null hypothesis for Friedman’s test is rejected, that is, there is a significant difference among the compared models, a post-hoc test is required to carry out pairwise comparisons. In this study, Fisher’s Least Significant Difference (LSD) was applied as a tool by which to identify the models that were statistically different in order to determine the optimal configuration [54]. The applied procedure is represented in Figure 4 for the SVM-RBF model as an example. In the case of SVM-POL, the procedure is made similar by replacing the value of γ with the polynomial degree value.

Once the optimal structure was defined for each configuration, SVM-POL and SVM-RBF, the results were compared with those obtained using traditional techniques such as the K-nearest neighbours (KNN) [55] and classification tree [56].

## 4. Results

The obtained results from the different configurations proposed for the SVM models are shown below. In this case, the influence of two different kernel functions was analysed: polynomial (SVM-POL) and radial basis functions (SVM-RBF). Furthermore, the influence of the regularisation parameter, C, was considered for both functions, with the aim of determining the optimal configuration of the proposed SVM model in this application. The values considered for the regularisation parameter were C = 2^0^, 2^1^,…, 2^8^; while for the polynomial kernel, the linear, quadratic, and cubic orders were evaluated. For the gamma parameter, the following values were tested: γ = 2^0^, 2^1^,…, 2^8^.

**Figure 5:**
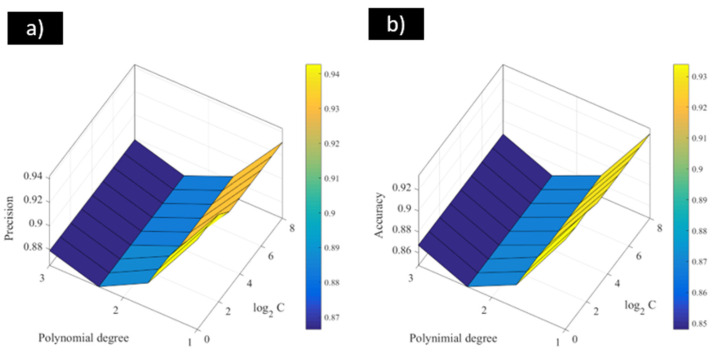
(**a**) Precision and (**b**) accuracy results of SVM-POL for modelling the localised corrosion of stainless steel in biogas environments.

Figure 5 shows the results in terms of the precision and accuracy obtained for the SVM-POL models. According to the figure, it can be seen that these models presented similar behaviours for both indices. In this case, the models that were the optimal ones for the localised corrosion modelling of stainless steel in biogas environments were SVM models considering the linear kernel function. For these models, the regularisation parameter had no great influence on the classification performance. The highest values related to the precision and accuracy indices provided by the SVM-POL model were 0.94 and 0.93, respectively. These values reflected the capacity of the proposed SVM-POL model to accurately predict the corrosion status of stainless steel after electrochemical testing in biogas environments.

**Figure 6:**
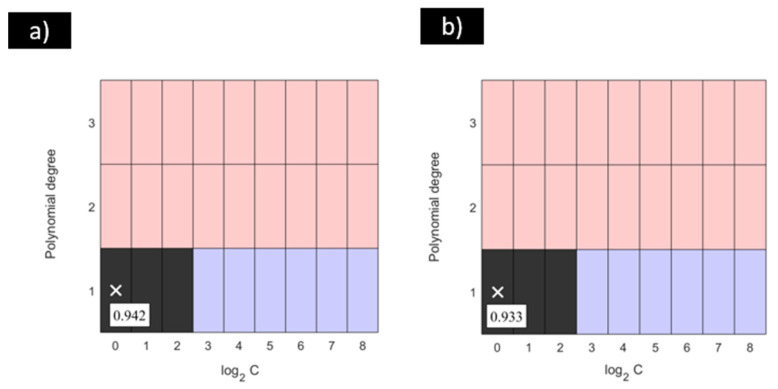
(**a**) Precision and (**b**) accuracy results of Friedman and Fisher LSD tests with a significance level of α = 0.05 for SVM-POL proposed for the model pitting and crevice corrosion of stainless steel in biogas environments.

In order to identify the optimal configurations for these models, a statistical procedure for multiple group comparison, considering precision and accuracy values, was applied according to the procedure shown in Figure 4. Firstly, the optimal values of C for each degree of the polynomial function considered in the SVM-POL models were identified. These models are represented in Figure 6 in a red colour. Secondly, the optimal degrees of the polynomial function for each C value were identified. These models are represented in Figure 6 in a blue colour. Comparing the results obtained from these steps, the models that were identified as optimal models for both steps are represented with grey colour. Finally, these models were subjected to a multiple comparison test with the aim of defining the optimal global equivalent configuration for the SVM-POL models presented in this study. As a result of the application of this statistical procedure, the optimal configurations of the SVM-POL model are represented in Figure 6 in a black colour. These configurations marked in a black colour were determined to be equivalent to the model that provided the highest values of precision and accuracy, at 0.94 and 0.93, respectively, marked with a cross in each graph. According to Figure 6, the optimal configurations for the SVM-POL model were determined to be the linear kernel function with C = 2^0^, 2^1^, 2^2^.

**Figure 7:**
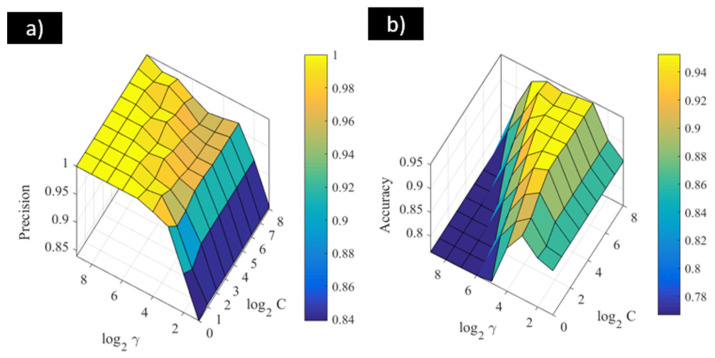
(**a**) Precision and (**b**) accuracy results of SVM-RBF for modelling the localised corrosion of stainless steel in biogas environments.

Similarly, the influence of the parameters involved in the SVM-RBF model was analysed. For this model, the results in terms of precision and accuracy are represented in Figure 7, where the influence of the C and γ values can be seen. According to Figure 7, the highest precision value reached by this model was 1. In this case, it can be observed that higher values of γ provided better results for the classification problem, whereas the regularisation parameter defined by C did not exhibit the same behaviour. For this case, as this parameter increased, a decrease in the classification performance was observed. This behaviour was different when the performance of the model in terms of accuracy was analysed. For accuracy results, the model provided the best classification performance when γ took intermediate values from the considered range, whereas the highest values of C provided the maximum value of accuracy, equal to 0.95.

**Figure 8:**
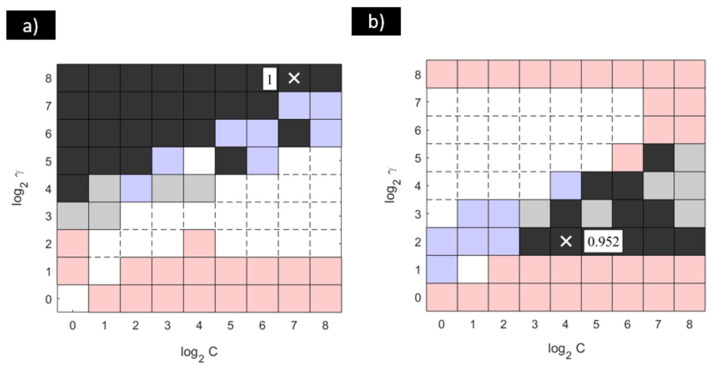
(**a**) Precision and (**b**) accuracy results of Friedman and Fisher LSD tests with a significance level of α = 0.05 for the SVM-RBF proposed to model the pitting and crevice corrosion of stainless steel in biogas environments.

With the aim of determining the optimal equivalent configurations that provided the best results for the SVM-RBF model, the procedure represented in Figure 4 was applied. The results obtained from the application of multiple comparison tests are collected in Figure 8.

According to the results collected in Figure 8, the equivalent configurations that provided the optimal precision value (precision = 1 in Figure 8a) were determined to be the SVM-RBF model with the following configurations; see Figure 8a: (γ = 2^4^–C = 2^0^), (γ = 2^5^–C = 2^0^, 2^1^, 2^2^, 2^5^), (γ = 2^6^–C = 2^0^, 2^1^, 2^2^,2^3^, 2^4^, 2^7^), (γ = 2^7^–C = 2^0^, 2^1^, 2^2^,2^3^, 2^4^, 2^5^, 2^6^), and (γ = 2^8^–C = 2^0^, 2^1^, 2^2^,2^3^, 2^4^, 2^5^, 2^6^, 2^7^, 2^8^). Related to the accuracy results, as seen in Figure 8b, the equivalent configurations providing the optimal behaviour (accuracy = 0.952) were determined to be the SVM-RBF model with the following pairs of values: (γ = 2^2^–C = 2^3^, 2^4^, 2^5^, 2^6^, 2^7^, 2^8^), (γ = 2^3^–C = 2^4^, 2^6^, 2^7^), (γ = 2^4^–C = 2^5^, 2^6^), and (γ = 2^5^–C = 2^7^).

Related to the results provided by the SVM-RBF model, it can be seen that there were different configurations providing the highest precision value. However, these configurations may be different from the configurations identified as the optimal ones when the accuracy results were analysed. This behaviour can be explained according to Equation (7). Based on this equation, for those cases where the models provided a precision value equal to 1, the false positive term results were null. This means that the model presented a high capacity to determine patterns that would not suffer localised corrosion. However, this model may not present a similar capacity to detect all of the patterns that will experience such an attack accurately, as no information about false negative patterns was included in terms of precision. For the optimal configurations of the SVM-RBF model in terms of accuracy, the maximum value reached was 0.952, which was much lower than the result obtained for precision.

With the aim of determining the optimal configurations for the proposed SVM models, SVM-POL and SVM-RBF, and looking for a balance between the capacity to detect the patterns that will suffer corrosion and those that will not suffer corrosion in biogas environments, the ROC space was applied.

The ROC space is created by representing the true positive rate (TPR), defined by Equation (9), versus the false positive rate (FPR), defined by Equation (10), for each configuration. As previously introduced, these measures can be computed from the confusion matrix for each classification model. The TPR corresponds to sensitivity, whereas the FPR is equivalent to 1-specificity. This graphic represents a useful tool by which to compare the classification performance of different models, as in these classification problems, the goal is to identify those models that provide acceptable discriminability between the existing classes: corrosion and no-corrosion patterns. This graphic is a two-dimensional graph that provides a trade-off between benefits (TP) and costs (FP). Each model can be represented by a single point in the ROC space, where the point (0, 1) represents the perfect classification. In this way, one model represented in the ROC space is better than another if it is to the northwest of the graphic [57].

In the following figure, the optimal configurations identified for the SVM models are represented, in addition to the developed model considering traditional classification techniques, such as classification tree (CT) and k-nearest neighbour (kNN), and considering three different values for k: 1, 3, and 5.

**Figure 9:**
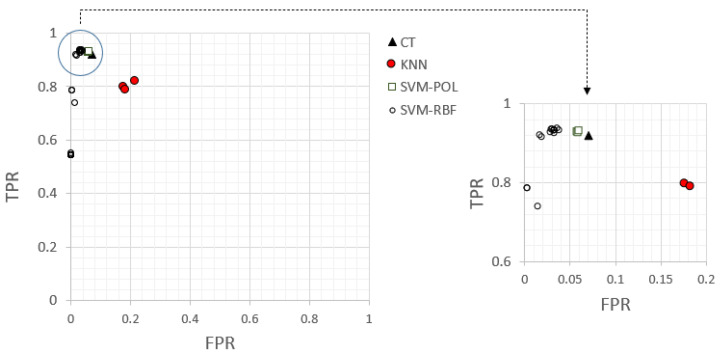
ROC graph showing the classification performance for the optimal configurations of the SVM models compared with traditional techniques: SVM-POL, represented by white squares; SVM-RBF, represented by white circles; CT, represented by a single, black triangle, and KNN, represented by red circles for the three values of k: 1, 3, and 5.

According to Figure 9, it can be seen that the CT provided better results than the kNN models. However, the CT presented lower efficiency than the SVM models. Therefore, the proposed SVM models become an efficient alternative to traditional techniques for this application. Specifically, there were some configurations for the SVM models that provided excellent specificity results (100%). These models are represented on the *Y*-axis (false positive = 0). However, these configurations were not considered to be the optimal ones, as they did not present a high capacity to detect all of the corrosion patterns correctly, as the number of FN patterns provided by these configurations was high. This is the reason why they are represented far from the upper left corner. For the application considered in this study, with the aim of finding a balance between the right classification of patterns that will suffer corrosion and those that will not suffer, the best configuration for the SVM model can be defined as the model located nearest the upper left corner. In this case, the optimal configuration for the SVM model was SVM-RBF (C = 2^6^ and γ = 2^3^), with sensitivity and specificity values of 94.0% and 96.6%, respectively. These values reflected the high capacity of the proposed model to predict the corrosion status of different grades of stainless steel in biogas environments without the need to perform microscopic analysis of the material surface, avoiding subjectivity in the results.

**Figure 10:**
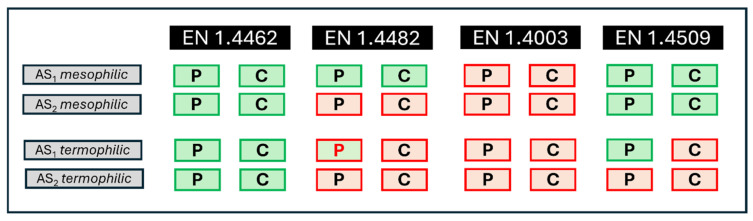
Comparison of experimental and predicted corrosion behaviours for selected stainless steel grades under different conditions with SVM-RBF (C = 2^6^ and γ = 2^3^).

According to this model, Figure 10 depicts the behaviour of each steel type under the analysed conditions, considering two different temperatures and two artificial solutions. In this figure, P corresponds to the pitting corrosion status, while C refers to the crevice corrosion status. The border colour of each rectangle indicates the experimental results: a red border signifies that the sample suffered corrosion, whereas a green border indicates that no corrosion was experimentally observed. The background colour of each rectangle represents the prediction provided by the model, with green indicating that the model predicts no corrosion and red indicating that corrosion is expected. In cases where the prediction does not match the experimental results, the corresponding letter (P or C) is displayed in red to highlight the discrepancy.

The proposed SVM model predicts the corrosion status of the material under previously untested experimental conditions. After performing electrochemical tests, the model can determine the surface condition without the need for microscopic analysis. Compared to manual classification, the model provides a significantly faster assessment while reducing subjectivity. Detecting pits or crevices after electrochemical testing is often challenging and may vary based on the laboratory analyst’s judgement, making the automated approach more reliable and consistent.

## 5. Conclusions

In this study, SVM models were proposed in order to determine the corrosion status of different grades of stainless steel used in biogas production. The classification performance of this technique was compared with traditional techniques, such as classification tree and k-nearest neighbour. Two different functions were analysed as kernel functions for SVM models: polynomic and RBF kernel functions. In this case, the maximum values of precision and accuracy were determined to be 100% and 95%, respectively. However, in order to evaluate the prediction performance of the proposed models, the limitations of the diagnostics based on these terms required the use of specificity and sensitivity terms. These indices are more meaningful than accuracy and precision and can be used to obtain the ROC space. According to the ROC space, it can be concluded that the SVM models outperformed the models based on traditional techniques. The optimal configuration for the SVM model was SVM-RBF (C = 2^6^ and γ = 2^3^), which provided values of sensitivity and specificity equal to 0.94 and 0.966, respectively. These results demonstrated the utility of the proposed SVM model to predict the corrosion status of stainless steel when localised corrosion (pitting and crevice corrosion) is analysed in biogas environments. The model can be presented as a complementary tool to electrochemical tests, as it predicts the corrosion status of different grades of stainless steel, according to the conditions involved in biogas production, with no need to analyse the surface microscopically.

## Figures and Tables

**Table 1:** Stainless steel grade compositions for experimental tests.

Stainless Steel Grade	Cr (%)	Mo (%)	N (%)	Mn (%)	Ti (%)	Nb (%)
EN 1.4404	16.71	2.018	0.038	1.291	0.016	0.016
EN 1.4462	22.892	3.279	0.1619	1.397	0.034	0.011
EN 1.4482	19.94	0.223	0.1374	4.016	0.025	0.006
EN 1.4003	11.45	0.01	0.0115	0.547	0.015	0.006
EN 1.4571	16.801	2.066	0.0134	1.578	0.301	0.008
EN 1.4509	18.492	0.051	0.0235	0.43	0.148	0.393
EN 1.4521	18.555	1.999	0.024	0.507	0.127	0.416
EN 1.4318	17.744	0.089	0.144	1.258	0.002	0.007

**Table 2:** Artificial solution conditions.

Reagent	g/L	Artificial Solution 1	Artificial Solution 2
Sodium Sulphate, Na_2_SO_3_	12.67	X	X
Ammonium Chloride, NH_4_Cl	69.81	X	X
Ammonium carbonate, (NH4)_2_CO_3_	15.13	X	X
Sodium Acetate Trihydrate, NaCH_3_COOH 3H_2_O	17.06	X	X
Hydrogen chloride, HCl, from FeCl_2_ desulfuration	7.31	--	X
pH range		8.2–8.5	6.6–7.2

**Table 3:** Details of the experimental conditions analysed for the electrochemical tests.

Experimental Conditions	Parameters
Material	Stainless steel
Grades	8 different grades of stainless steel
Solutions	Two types: AS1/AS2
Temperature	35 °C (mesophilic)/50 °C (thermophilic)
Number of tests per condition	3
Electrochemical test	Pitting/crevice corrosion tests
Surface finish	No polishing/#600# grain polishing

## Data Availability

The data presented in this study are available on request from the corresponding author.
